# DPPA3 facilitates genome-wide DNA demethylation in mouse primordial germ cells

**DOI:** 10.1186/s12864-024-10192-7

**Published:** 2024-04-05

**Authors:** Keisuke Toriyama, Wan Kin Au Yeung, Azusa Inoue, Kazuki Kurimoto, Yukihiro Yabuta, Mitinori Saitou, Toshinobu Nakamura, Toru Nakano, Hiroyuki Sasaki

**Affiliations:** 1 Division of Epigenomics and Development, Medical Institute of Bioregulation, Kyushu University, Fukuoka, 812-8582 Japan; 2 Laboratory for Epigenome Inheritance, Riken Center for Integrative Medical Sciences, Kanagawa, 230-0045 Japan; 3 Tokyo Metropolitan University, Tokyo, 192-0397 Japan; 4 Department of Embryology, School of Medicine, Nara Medical University, 840 Shijo-Cho, Kashihara, Nara 634-8521 Japan; 5 Institute for the Advanced Study of Human Biology (ASHBi), Kyoto University, Yoshida-Konoe- cho, Sakyo-ku, Kyoto, 606-8501 Japan; 6 Department of Anatomy and Cell Biology, Graduate School of Medicine, Kyoto University, Yoshida-Konoe-cho, Sakyo-ku, Kyoto, 606-8501 Japan; 7 Center for iPS Cell Research and Application (CiRA), Kyoto University, 53 Kawahara-cho, Shogoin, Sakyo-ku, Kyoto, 606-8507 Japan; 8 Laboratory for Epigenetic Regulation, Department of Animal Bio-Science, Nagahama Institute of Bio-Science and Technology, Shiga, 526-0829 Japan; 9 Graduate School of Frontier Biosciences, Osaka University, Osaka, 565-0871 Japan

**Keywords:** Stella, Dppa3, Prdm14, Tet1, Primordial germ cell, DNA demethylation, Epigenetic reprogramming

## Abstract

**Background:**

Genome-wide DNA demethylation occurs in mammalian primordial germ cells (PGCs) as part of the epigenetic reprogramming important for gametogenesis and resetting the epigenetic information for totipotency. *Dppa3* (also known as *Stella* or *Pgc7*) is highly expressed in mouse PGCs and oocytes and encodes a factor essential for female fertility. It prevents excessive DNA methylation in oocytes and ensures proper gene expression in preimplantation embryos: however, its role in PGCs is largely unexplored. In the present study, we investigated whether or not DPPA3 has an impact on CG methylation/demethylation in mouse PGCs.

**Results:**

We show that DPPA3 plays a role in genome-wide demethylation in PGCs even before sex differentiation. *Dppa3* knockout female PGCs show aberrant hypermethylation, most predominantly at H3K9me3-marked retrotransposons, which persists up to the fully-grown oocyte stage. DPPA3 works downstream of PRDM14, a master regulator of epigenetic reprogramming in embryonic stem cells and PGCs, and independently of TET1, an enzyme that hydroxylates 5-methylcytosine.

**Conclusions:**

The results suggest that DPPA3 facilitates DNA demethylation through a replication-coupled passive mechanism in PGCs. Our study identifies DPPA3 as a novel epigenetic reprogramming factor in mouse PGCs.

**Supplementary Information:**

The online version contains supplementary material available at 10.1186/s12864-024-10192-7.

## Background

DNA methylation in the mammalian genome occurs predominantly in the context of CpG dinucleotide (CG methylation) and converts cytosine to 5-methylcytosine (5mC). It is important for many biological processes, including development, transposon silencing, genomic imprinting, and X-chromosome inactivation [1]. During development, lineage-specific CG methylation patterns are established and then maintained in somatic cells, but genome-wide demethylation occurs as part of the epigenetic reprogramming in germ cells and early embryos [2–4].

Primordial germ cells (PGCs), the common precursors of sperms and oocytes, arise in the epiblast by embryonic day 7.0 (E7.0) of mouse development. They undergo epigenetic reprogramming, including genome-wide CG demethylation, the first phase of which likely occurs via a replication-coupled passive mechanism [5–7]. Imprinting control regions (ICRs), X-linked CpG islands, and germline genes are relatively resistant to this demethylation [6, 8]. The remaining 5mC is progressively converted to 5-hyroxymethylcytosine (5hmC), most predominantly during E10.5–E11.5, via the action of Ten-eleven translocation methylcytosine dioxygenase 1 (TET1) [9–11], and then replaced by cytosine via either a passive mechanism or base excision repair [12]. By E13.5, the PGC genome, including the ICRs and germline genes but excluding some retrotransposons, becomes almost fully hypomethylated [6, 13]. Thus, the second phase of demethylation mediated by Tet1 is likely required for full reprogramming of the entire genome, except for the retrotransposons [11, 14, 15]. Female germ cells then maintain a hypomethylated state until the non-growing oocyte stage [16].

DPPA3 (also known as STELLA or PGC7) is a small protein produced in mouse PGCs and oocytes; gene knockout (KO) studies have revealed that, while KO mice develop normally, KO females are infertile. It was revealed that *Dppa3* serves as a maternal effect gene essential for preimplantation development [17–19]. While previous studies have reported a possible role for DPPA3 in protecting the maternal genome from TET-mediated CG demethylation [20–22], more recent studies have paradoxically indicated its role in demethylation or the prevention of excess methylation [23–26]. In contrast, DPPA3’s role in PGCs has not been explored well, partly due to the lack of a phenotype in KO PGCs [19, 27]. However, DPPA3 does exist in the nucleus of PGCs and then relocates to the cytoplasm after E10.5 [27], and a polymerase chain reaction (PCR)-based, sequence-specific methylation assay revealed the hypermethylation of certain retrotransposons in *Dppa3* KO PGCs [27].

In the present study, we investigated whether or not DPPA3 has a broader impact on CG methylation/demethylation in PGCs by whole-genome bisulfite sequencing (WGBS). We also investigated how the effect is brought about by examining the involvement of some regulatory pathways known to have a role in epigenetic reprogramming in this particular cell type.

## Results

### CG hypermethylation occurs in *Dppa3* KO PGCs

**Fig. 1:**
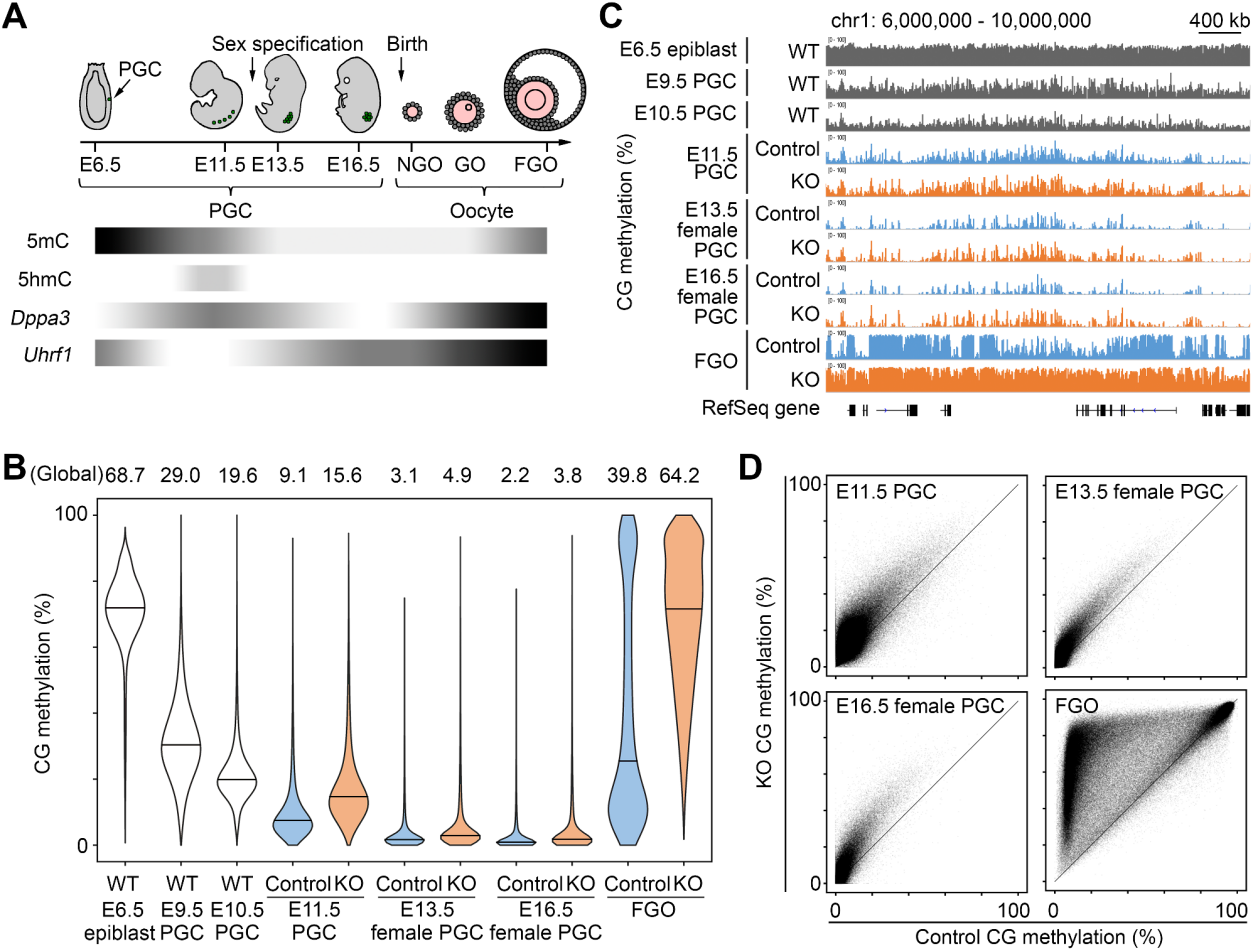
CG methylation reprogramming is partially impaired in *Dppa3*KO PGCs. (**A**) Chronology of PGC development and oocyte growth and changes in 5mC and 5hmC levels and *Dppa3* and *Uhrf1* expression. NGO, non-growing oocyte; GO, growing oocyte; FGO, fully grown oocyte. (**B**) Violin plots showing the distributions of regional CG methylation levels (in 10-kb windows) in control and KO PGCs and FGOs (only windows informative in all samples were used, *n* = 249,088). Published WGBS data [6, 11] were retrieved and reprocessed for wild-type epiblasts, E9.5 PGCs, and E10.5 PGCs. Horizontal bars indicate the median values. The global CG methylation levels are indicated above the plots. (**C**) CG methylation profiles of a portion of mouse chromosome 1. RefSeq genes are indicated at the bottom. (**D**) Scatterplots comparing regional CG methylation levels (10-kb windows, *n* = 249,088) in control and KO PGCs and FGOs

To investigate the role of DPPA3 in epigenetic reprogramming of PGCs, we used previously reported *Dppa3* KO mice, which carried a reverse tetracycline-transactivator insertion causing premature transcription termination [20]. A transgene encoding an enhanced green fluorescent protein (EGFP) driven by the *Pou5f1* promoter (*Pou5f1*-*Egfp*), of which expression marks PGCs [28], was introduced by crossing. We collected control and *Dppa3* KO PGCs from *Dppa3*^+/−^ and *Dppa3*^−/−^ embryos at E11.5 (sex differentiation not yet evident at this stage) (Fig. 1A, Additional file 1: Fig. S1A and Table S1) and subjected them to WGBS as described [29, 30]. In the subsequent stages, we focused on female germ cells and collected PGCs from female gonads at E13.5 and E16.5 (Fig. 1A, Additional file 1: Fig. S1A and Table S1) and fully grown oocytes (FGOs) from adult ovaries after postnatal 10 weeks. Following confirmation of the reproducibility in biological replicates (500-kilobase [kb] windows, *R* = 0.93$\sim$0.99), data from these scarce cells were combined for downstream analyses (Additional file 1: Table S2).

The global CG methylation levels of heterozygous control PGCs were very low at E11.5–16.5 (9.1–2.2%) and also consistent with the ongoing CG demethylation in wild-type PGCs (Additional file 1: Fig. S1B) [6, 11, 31]. *Dppa3* KO PGCs had CG methylation levels 1.5- to 1.8-fold higher than those of control PGCs, although the actual differences were small (ΔCG methylation 6.5%, 1.8%, and 1.6% at E11.5, E13.5, and E16.5, respectively) (Fig. 1B). The overall CG methylation patterns (in 10-kb windows) were grossly similar between the genotypes (Fig. 1C), but there were regions showing greater hypermethylation (Fig. 1D). These results suggest that the hypermethylation previously discovered at selected repetitive elements [27] extends to the whole genome and that DPPA3 plays a wider role in CG demethylation of PGC. Importantly, since the PGCs from *Dppa3* KO mice proliferate normally [19, 27], the observed hypermethylation is not attributable to a reduced dilution rate due to slower DNA replication.

Female germ cells undergo *de novo* CG methylation during the growing oocyte stage in the postnatal ovary (Fig. 1A) [16, 32]. The global CG methylation level of heterozygous control FGOs was similar to that of wild-type FGOs (39.8% vs. 39.2%) (Additional file 1: Fig. S1B), but *Dppa3* KO FGOs showed a global CG methylation level 1.6-fold higher than the control FGOs (64.2%, ΔCG methylation 24.4%) (Fig. 1B). While previous works identified hypermethylated gene promoters and CpG-rich regions in *Dppa3* KO FGOs [24, 33], our WGBS clearly extended their observations to broader regions normally hypomethylated in FGOs, including intergenic regions (Fig. 1C,D).

### IAP elements marked with H3K9me3 are the most hypermethylated in *Dppa3* KO PGCs

**Fig. 2:**
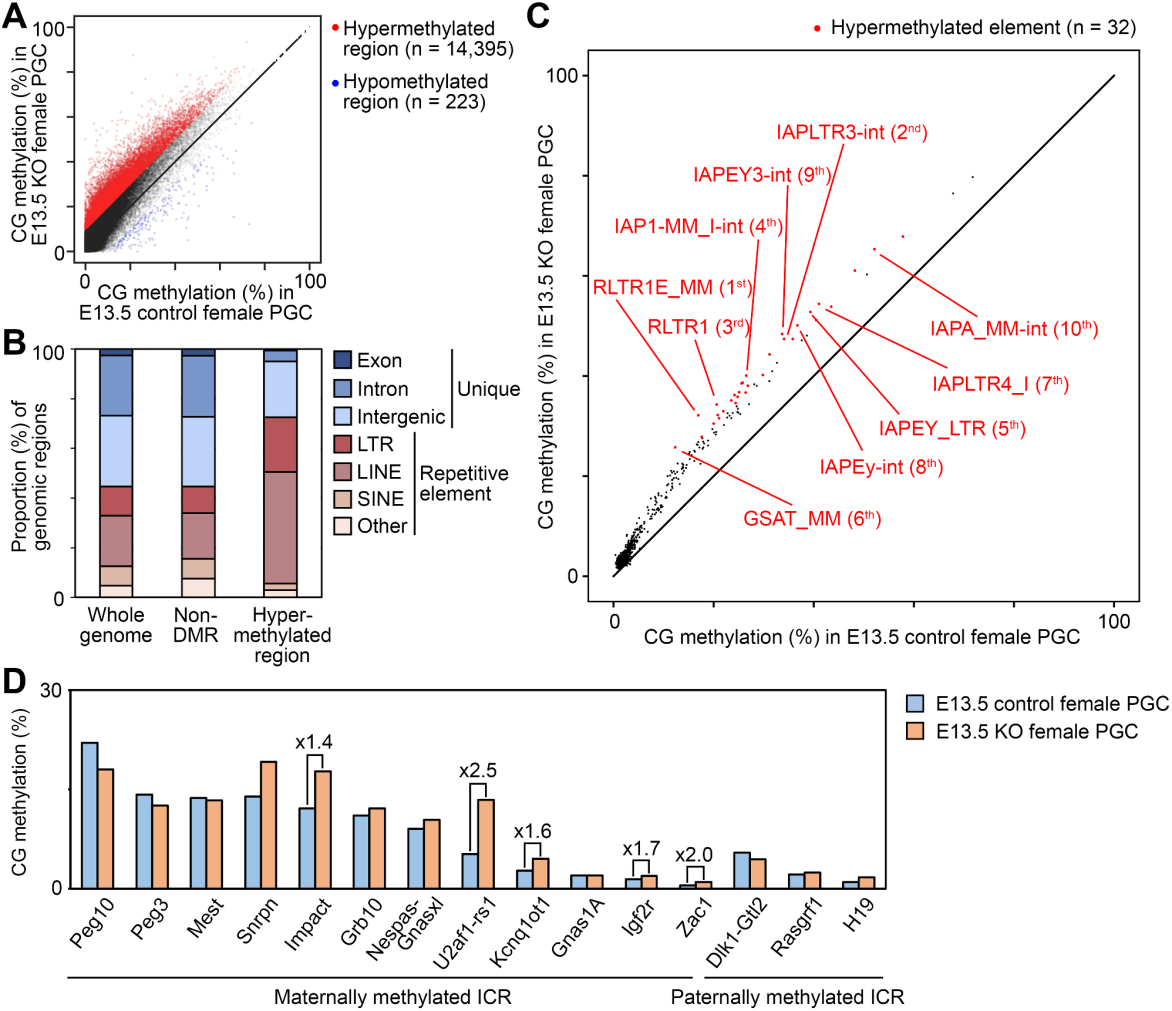
Hypermethylated regions are enriched for specific repetitive elements. (**A**) Scatterplots comparing regional CG methylation levels (10-kb windows) in E13.5 control and KO female PGCs (*n* = 254,013). Hypermethylated (ΔCG methylation > 10%, *n* = 14,395) and hypomethylated (ΔCG methylation <–10%, *n* = 223) regions are highlighted in red and blue, respectively. (**B**) Genomic context of the regions hypermethylated in E13.5 KO female PGCs. The proportions of indicated context categories relative to the total size of the hypermethylated regions (143.95 Mb) are shown. The genomic contexts of the whole genome and regions not showing differential methylation (non-DMRs) are also shown for comparison. (**C**) Scatterplots comparing CG methylations levels of repetitive elements in E13.5 control and KO female PGCs (*n* = 888). Only those with ≥ 50 genomic copies were analyzed. Hypermethylated elements (*n* = 32) are highlighted in red, and the top 10 are labelled with their respective rankings. (**D**) CG methylation levels at the ICRs in E13.5 control and KO female PGCs. The ICRs showing > 1.3-fold increase in CG methylation are marked with actual fold increase values

We then attempted to identify genomic regions hypermethylated in E13.5 *Dppa3* KO female PGCs. There were 14,618 differentially methylated regions (10-kb windows, ΔCG methylation > 10%), of which 14,395 were hypermethylated and 223 hypomethylated (Fig. 2A). This hypermethylation was also observed in E11.5 KO PGCs, E16.5 KO female PGCs and KO FGOs (Additional file 1: Fig. S2A). The hypermethylated regions were enriched for retrotransposons, such as the long terminal repeat (LTR) elements and long interspersed nuclear elements (LINEs) (1.9- and 2.2-fold enrichment over the whole genome, respectively) (Fig. 2B). Among the 888 repetitive element families including retrotransposons and tandem repeats, 32 were hypermethylated (ΔCG methylation > 10%), and 7 of the top 10 were intracisternal A particle (IAP) elements, a class of relatively young and active LTR elements (Fig. 2C). One LINE element (L1Md_F) was among the 32 hypermethylated elements, but the extent of its hypermethylation was smaller than that of IAP elements. These results are consistent with the previous locus-specific study on IAP and LINE-1 elements [27]. Interestingly, one of the top 10 was the major satellite repeat (GSAT_MM) (Fig. 2C), which was previously reported to be hypermethylated in *Dppa3* KO GOs [24]. This is consistent with the enhanced 5mC staining of DAPI-dense chromatin that we observed in *Dppa3* KO GOs (Additional file 1: Fig. S2B). Since the GOs of this stage are just at the beginning of *de novo* DNA methylation (Additional file 1: Fig. S2C), the hypermethylation likely persisted from the PGC stage. In addition, one-third of the ICRs (5/15), of which allele-specific methylation is normally erased by E13.5 [6, 11, 15], were also hypermethylated (> 1.3-fold) in *Dppa3* KO PGCs (the *Impact*, *U2af1-rs1*, *Kcnq1ot1*, *Igf2r*, and *Zac1* ICRs) (Fig. 2D).

**Fig. 3:**
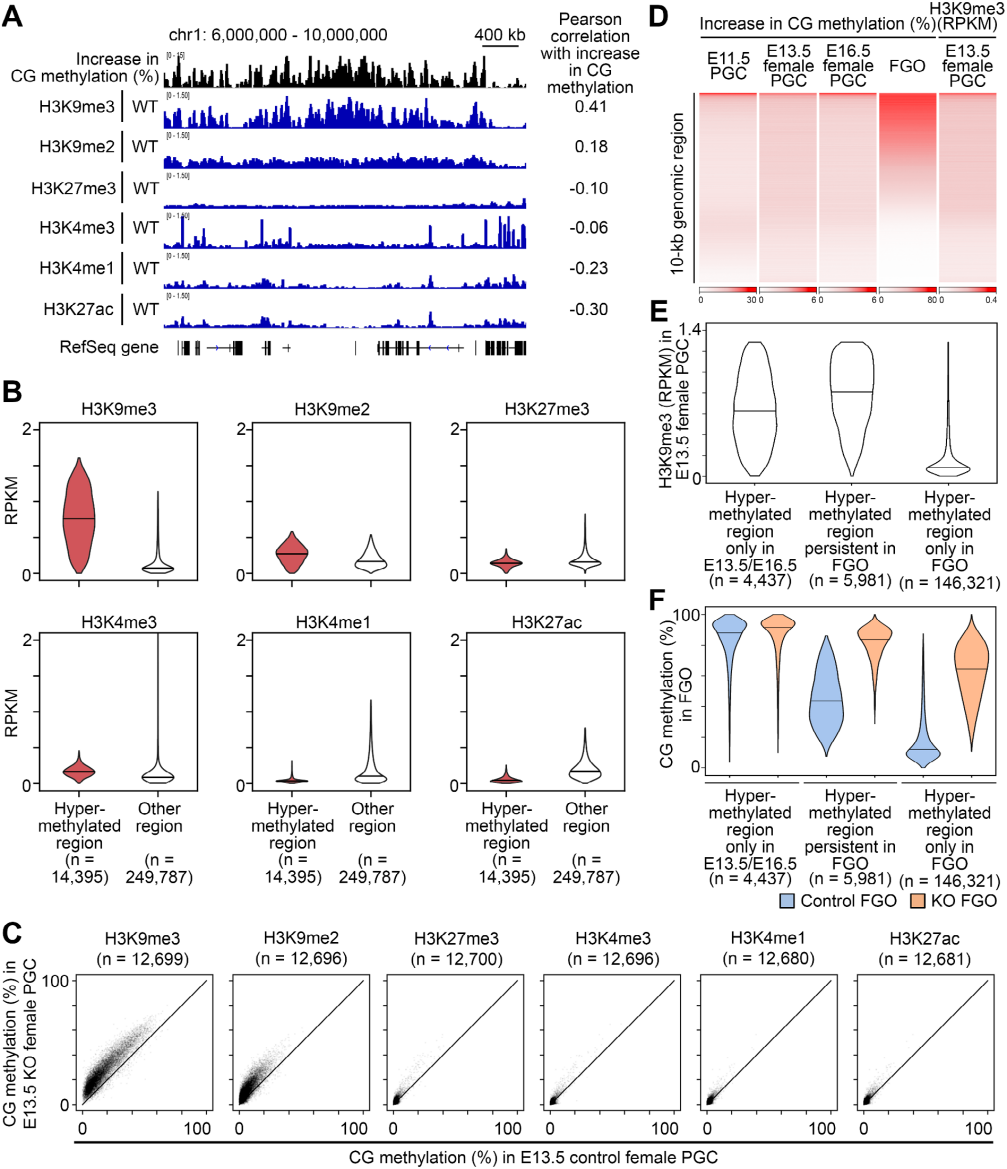
Hypermethylated regions are enriched for H3K9me3. (**A**) Increase in CG methylation (%) (KO– control) and histone mark profiles of a portion of mouse chromosome 1 in E13.5 wild-type female PGCs. Published ChIP-seq data [34] were retrieved and reprocessed. RefSeq genes are indicated at the bottom. (**B**) Violin plots showing the enrichment of histone marks in the hypermethylated (10-kb windows, *n* = 14,395) and other regions (*n* = 249,787) regions in E13.5 wild-type female PGCs. Horizontal bars indicate the median values. (**C**) Scatterplots comparing CG methylation levels of regions with specific histone marks (10-kb windows) in control and KO E13.5 female PGCs. Top 5% genomic regions with enrichment for specific histone marks were analyzed. The actual numbers of used windows are indicated for individual graphs. (**D**) Heatmap showing regional increase in CG methylation (%) in E11.5–16.5 PGCs and FGOs. H3K9me3 enrichment is from wild-type E13.5 female PGCs. (**E**) Violin plots showing the H3K9me3 enrichment in E13.5 wild-type female PGCs for regions hypermethylated only in E13.5/E16.5, persistent in FGO and only in FGO (identified in Additional file 1: Fig. S3F). Horizontal bars indicate the median values. (**F**) Violin plots showing the regional CG methylation levels in control and KO FGOs for regions hypermethylated only in E13.5/E16.5, persistent in FGO and only in FGO. Horizontal bars indicate the median values

We then searched for a link between the CG hypermethylation and histone marks. We reprocessed published ChIP-seq data on various histone marks in wild-type E13.5 female PGCs [34] and found that the aberrant hypermethylation was most closely associated with histone H3 lysine-9 tri-methylation (H3K9me3) (Fig. 3A,B,C). This is consistent with the above finding that LTR elements, especially IAP elements, were the most severely affected, as these elements are marked and silenced by H3K9me3 in PGCs [35]. Previous immunostaining also showed that the pericentromeric heterochromatin (which includes the major satellite repeat) is marked by H3K9me3 in E11.5 and E13.5 PGCs [36]. In contrast, regions marked by H3K4me3, H3K4me1, H3K27ac, and H3K27me3, which are found in active regulatory elements and genic regions, were not associated with hypermethylation (Fig. 3B,C). The aberrant retention of CG methylation in the H3K9me3-marked regions is interesting, as this mark is recognized by the CG methylation maintenance factor ubiquitin-like with PHD and ring finger domain 1 (UHRF1) [37], which is released from the chromatin [23, 25], or even exported out of the cell nucleus [24, 26], by the action of DPPA3 in other cell types.

### *Dppa3* KO has little impact on gene expression in PGCs

To investigate the impact of *Dppa3* KO and CG hypermethylation on gene expression, we next performed RNA-seq with E13.5 control and KO female PGCs (Additional file 1: Table S3). Only two genes were significantly affected (fold change > 4, *p* < 0.05): one was *Dppa3*, of which downregulation confirmed the genetic ablation, and the other was the choline kinase beta gene (*Chkb*), which showed aberrant derepression (Additional file 1: Fig. S3A). While loss-of-function mutations in human *CHKB* are associated with muscular dystrophy [38], the impact of its ectopic expression is currently unknown. The expression of repetitive elements was unchanged in KO PGCs (Additional file 1: Fig. S3B). In addition, all DNA methyltransferases (*Dnmt1*, *Dnmt3a*, *Dnmt3b*, *Dnmt3c*, and *Dnmt3l*) and *Uhrf1* remained unaffected (Additional file 1: Fig. S3C), suggesting that the observed hypermethylation was not due to their misregulation.

### Aberrant CG hypermethylation in PGCs persists in postnatal oocytes

Female PGCs maintain a globally hypomethylated state beyond E13.5, until *de novo* methylation starts after birth in GOs [16, 32]. We traced the fate of the aberrant CG hypermethylation (ΔCG methylation > 10%) detected in *Dppa3* KO female PGCs up to the FGO stage (Fig. 3D). A small proportion (17%, *n* = 12,407) of the regions hypermethylated in E11.5 KO PGCs (*n* = 72,907) remained hypermethylated at E13.5 (*n* = 3,608), E16.5 (*n* = 4,781), or both (*n* = 4,018) (Additional file 1: Fig. S3D). Notably, these regions were marked by H3K9me3 at E13.5 (Additional file 1: Fig. S3E). The rest of the regions hypermethylated in E11.5 KO PGCs (83%, *n* = 60,500) became almost unmethylated (mean CG methylation 3.9%) and were devoid of H3K9me3, suggesting that a DPPA3-independent mechanism compensates for the demethylation failure after E11.5. In contrast, about half (51%, *n* = 7,346) of the regions hypermethylated in E13.5 PGCs (*n* = 14,395) remained hypermethylated in E16.5 PGCs (Additional file 1: Fig. S3D).

We then identified 168,120 regions showing altered methylation in KO FGOs, of which 165,899 were hypermethylated (ΔCG methylation > 10%) (Additional file 1: Fig. S3F). Strikingly, 82% (*n* = 11,818) and 81% (*n* = 13,741) of the regions hypermethylated in E13.5 (*n* = 14,395) and E16.5 KO PGCs (*n* = 16,966), respectively, were also hypermethylated in KO FGOs (Additional file 1: Fig. S3F). Furthermore, a total of 5,981 regions were persistently hypermethylated from E13.5 to the FGO stage (42% of those hypermethylated at E13.5 and 35% of those hypermethylated at E16.5). Lastly, a large fraction (69%, *n* = 50,543) of the regions hypermethylated in E11.5 KO PGCs (*n* = 72,907) was also hypermethylated in KO FGOs. The major satellite repeat remained hypermethylated as well (Additional file 1: Fig. S3G), consistent with the previous observations in GOs and metaphase II oocytes [24]. Taken together, these findings suggest that once the hypermethylated state survives through the active demethylation phase between E11.5 and E13.5, it preferentially remains to be hypermethylated up to the FGO stage, although KO FGOs gain a large number of newly hypermethylated regions.

When we sought for the chromatin features of the regions persistently hypermethylated from E13.5 to the FGO stage, they tended to have high levels of H3K9me3 at E13.5 (Fig. 3D,E). Notably, the persistently hypermethylated regions had CG methylation levels higher than those of the FGO-specific hypermethylated regions in control FGOs (Fig. 3F). Regions hypermethylated in E13.5 and E16.5 PGCs (*n* = 4,437) were almost fully methylated in both control and KO FGOs (Fig. 3F).

It was previously reported that the CG hypermethylation of *Dppa3* KO FGO in part persists to the two-cell-embryo stage and causes misregulation of genes [24]. Of the 1,637 transcripts reported to be downregulated in maternal KO 2-cell embryos [24], 15 were located in the persistently hypermethylated regions and 862 in the FGO-specific hypermethylated regions. The 15 downregulated transcripts were not initiated from repetitive elements and a majority (11/15) belonged to the olfactory receptor family (Additional file 1: Table S4). Thus, the persistent hypermethylation initiating from E13.5 PGCs appears to impact at least some genes in two-cell embryos, but its significance in the developmental phenotype is currently unknown.

### DPPA3 is regulated by PRDM14 in PGCs

**Fig. 4:**
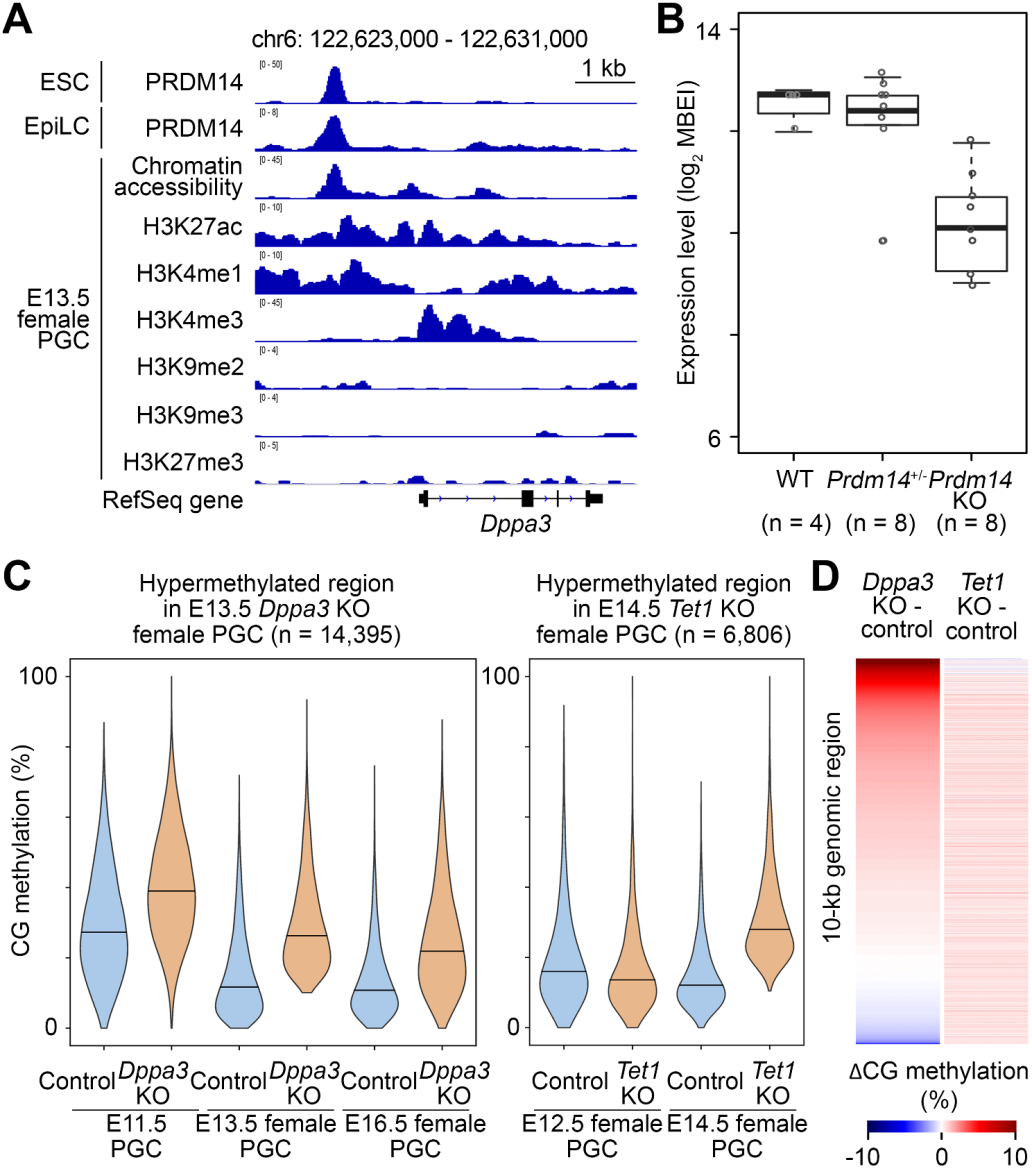
DPPA3 acts in the downstream of PRDM14 and independently of TET1. (**A**) ChIP-seq data showing a PRDM14 peak in an upstream region of *Dppa3* and enrichment of histone marks typical of an active enhancer. Chromatin accessibility data is also shown. Published ChIP-seq data from ESCs and EpiLCs overexpressing *Prdm14* [40, 41] and from E13.5 female PGCs [34] were reprocessed. (**B**) *Dppa3* expression in E7.25-E7.5 *Prdm14* KO PGCs revealed by single cell microarray analysis. (**C**) Violin plots showing that CG hypermethylation of *Dppa3* KO PGCs is already seen at E11.5 (10-kb windows, *n* = 14,395) (left) while hypermethylation of *Tet1* KO PGCs occurs only after E12.5 (*n* = 6,806) (right). Horizontal bars indicate the median values. Published WGBS data [11] were retrieved and reprocessed for control and *Tet1* KO female PGCs. (**D**) Heatmap showing that different regions are hypermethylated in *Dppa3* KO and *Tet*1 KO E13.5 female PGCs (*n* = 166,566). The heatmap was produced based on the extent of CG methylation differences (∆CG methylation). Published WGBS data [14] were retrieved and reprocessed for control and *Tet1* KO PGCs

PR domain-containing transcription factor 14 (PRDM14) is a master regulator of epigenetic reprogramming in embryonic stem cells (ESCs) and PGCs [39]. Given the observed role for DPPA3 in CG demethylation in PGCs, we wondered if there was a link between PRDM14 and DPPA3. Using published ChIP-seq data in mouse ESCs and epiblast-like cells (EpiLCs) expressing exogenous *Prdm14* [40, 41], we found a sharp PRDM14 peak in a region 1.8-kb upstream of *Dppa3*, with chromatin features typical of an active enhancer in E13.5 female PGCs [34] (Fig. 4A). Furthermore, *Dppa3* was downregulated in *Prdm14* KO PGCs [42], revealed by microarray and quantitative PCR analyses (Fig. 4B; Additional file 1: Fig. S4A). In addition, a reanalysis of our RNA-seq data from *Prdm14* KO PGC-like cells (PGCLCs) [43] showed a failure in *Dppa3* activation during their specification in vitro (Additional file 1: Fig. S4B). These results are consistent with the previously reported diminished immunostaining of DPPA3 in *Prdm14* KO PGCs [42]. Taken together, these data suggest that *Dppa3* is regulated by PRDM14, presumably through a direct mechanism, and works downstream of this master regulator of epigenetic reprogramming in PGCs.

### DPPA3 works independently of TET1 in CG demethylation in PGCs

It was previously reported that *Dppa3* KO PGCs have higher 5mC levels and lower 5hmC levels at both IAP and L1 elements than control PGCs [27]. As 5hmC is a demethylation intermediate generated from 5mC via the catalytic activity of TET dioxygenases [12], Nakashima et al. [27] speculated that DPPA3 may have a role in TET-mediated demethylation in PGCs. Among the *Tet* family genes, *Tet1* is the most highly expressed in PGCs (our reanalysis of published data from [6] and [44]; Additional file 1: Fig. S4C) and furthermore shown to be responsible for the majority of 5hmC detected in PGCs [11]. To test whether TET1 is involved in demethylation facilitated by DPPA3, we compared our WGBS and RNA-seq data from *Dppa3* KO PGCs with those from *Tet1* KO PGCs [11, 14]. It was found that, first, while *Dppa3* KO PGCs showed failure in demethylation already at E11.5, *Tet1* KO PGCs started to show it only after E12.5 [11] (Fig. 4C). Second, *Dppa3* and *Tet1* KO affected CG methylation of different genomic regions in E13.5 female PGCs [14] (Fig. 4D). Finally, while a total of 899 genes were downregulated in *Tet1* KO female PGCs at E13.5 (false discovery rate < 0.05) [14], their expression was totally unaffected in *Dppa3* KO PGCs. Conversely, *Chkb*, which showed aberrant derepression in *Dppa3* KO PGCs (Additional file 1: Fig. S3A), was unaffected in *Tet1* KO PGCs. In addition to the above, while a recent study showed that *Dppa3* is downregulated in *Tet1/Tet2* KO naïve embryonic stem cells [26], this does not occur in *Tet1* KO female PGCs [14]. These results strongly suggest that DPPA3 and TET1 work independently in CG demethylation in mouse PGCs.

## Discussion

Genome-wide epigenetic reprogramming is an important event in mammalian PGC development. In this study, we investigated the role of DPPA3 in the epigenetic reprogramming of PGCs using *Dppa3* KO mice and found that this small protein facilitates genome-wide CG demethylation even before sex differentiation. The study thus extends the previous observation that certain retrotransposons are hypermethylated in *Dppa3* KO PGCs [27]. Since it appears that DPPA3 works downstream of PRDM14, a master regulator of epigenetic reprogramming, it is likely that the DPPA3-dependent CG demethylation is part of more comprehensive epigenetic reprogramming orchestrated by this factor.

Regarding how DPPA3 facilitates CG demethylation in PGCs, we speculate that the mechanism involves UHRF1, a CG methylation maintenance factor [37]. Previous studies showed that DPPA3 disrupts the UHRF1 binding to the chromatin in cultured cells [23, 25] and even facilitate its export to the cytoplasm in oocytes and embryonic stem cells [24, 26]. Together with these and other findings, it has been suggested that, in non-proliferating oocytes, DPPA3 prevents aberrant de novo methylation mediated by DNMT1 and UHRF1 [24]. We envisage that DPPA3 perhaps facilitates replication-dependent passive demethylation in proliferating PGCs. The involvement of UHRF1 is consistent with the observation that the genomic regions aberrantly CG hypermethylated in *Dppa3* KO PGCs were marked with H3K9me3, as this protein contains a tandem Tudor domain recognizing H3K9me3 [45]. Furthermore, lines of evidence that we presented in this study suggest that DPPA3 contributes to the CG demethylation independently of TET1, the major enzyme that converts 5mC to 5hmC in PGCs (Hill et al. 2018; Additional file 1: Fig. S4C). In naïve embryonic stem cells, PRDM14 maintains the hypomethylated state of the genome through TET1/TET2-mediated active demethylation [46]. If this applies to PGCs as well, PRDM14 should regulate multiple CG demethylation pathways involving both active and passive mechanisms in these cells.

*Dppa3* is dispensable for PGC development [19, 27] but serves as a maternal effect gene essential for preimplantation development [17–19]. Previous works have indicated that, in oocytes that lack DPPA3, both the chromatin state and cytoplasmic factors acquire defects that impair the development of maternal KO embryos [24, 47]. Since the defects imposed during oogenesis conceal those originating from PGCs, it is difficult to know whether or not the loss of *Dppa3* in PGCs contributes to the developmental phenotype. However, our data show that at least some genes persistently hypermethylated in KO PGCs and FGOs are downregulated in maternal KO 2-cell embryos, leaving the possibility that they play a role in the developmental defect. Furthermore, a previous work indicated that maternal KO 2-cell embryos show impaired chromocenter formation, probably due to the downregulation of the H3.3-specific histone chaperone DAXX, followed by impaired H3.3 incorporation and reduced reverse-strand transcription of the major satellite repeat, which are essential for chromocenter formation [47]. The persistent CG hypermethylation of the major satellite initiated in KO PGCs could persist to embryos and contribute to the reduced transcription of this repeat. Further studies are needed to understand the precise role of DPPA3 in the reprogramming of DNA methylation in PGCs.

## Conclusions

Mouse DPPA3 plays a role in genome-wide DNA demethylation in E11.5 PGCs before sex differentiation. *Dppa3* knockout female PGCs therefore show aberrant hypermethylation at E13.5 and E16.5, most predominantly at H3K9me3-marked retrotransposons, which persists up to the FGO stage. DPPA3 works downstream of PRDM14, a master regulator of epigenetic reprogramming in embryonic stem cells and PGCs, and independently of TET1, an enzyme that hydroxylates 5-methylcytosine. The results suggest that DPPA3 facilitates DNA demethylation through a replication-coupled passive mechanism. Our study identifies DPPA3 as a novel epigenetic reprogramming factor in mouse PGCs.

## Methods

### Genetically modified mice and genotyping

Mice carrying an insertion at the first exon of *Dppa3* (*Dppa3* KO mice) and those carrying an *Pou5f1*-*Egfp* transgene were described previously [20, 28]. They were of the C57BL/6J background. Genotyping was performed by PCR using the primers described in the original reports.

### Embryo, PGC, and FGO collection

Embryos were obtained at E11.5, E13.5 and E16.5 from the uteri of *Dppa3*^+/−^ females crossed with *Dppa3*^−/−^ males carrying the *Pou5f1*-*Egfp* transgene. The sex of the embryos was determined by visual inspection of the gonads at E13.5 and E16.5. After genotyping for the *Dppa3* alleles and the transgene, the gonads were digested using 0.25% trypsin/0.5 mM EDTA/1 µg/ml DNaseI in phosphate-buffered saline (PBS) [48]. EGFP-positive PGCs were isolated using a FACSMelody system (BD Bioscience) into a low-retention 1.5-ml tube containing 0.1% bovine serum albumin (BSA) in PBS. After centrifugation, supernatant was removed. Embryos were also obtained at E7.25-E7.5 from the uteri of *Prdm14*^+/−^ females crossed with *Prdm14*^+/−^ males. After genotyping for the *Prdm14* alleles, the base of the allantois bud (PGC-enriched) was dissected and digested using 0.05% trypsin/0.5 mM EDTA in PBS. Single cells were randomly picked for cDNA synthesis. FGOs were harvested from adult (≥ 10 weeks old) ovaries by needle puncture. The cells were flash-frozen in liquid nitrogen and stored at − 80 °C until use.

### WGBS and RNA-seq

WGBS libraries were constructed using the post-bisulfite adaptor tagging method as described [29, 30]. PGCs and FGOs were spiked with 1% unmethylated lambda phage DNA (Promega). Libraries were amplified with KAPA library amplification kit (KAPA) for four cycles. For RNA-seq, total RNA was extracted from PGCs using Trizol reagent (Thermo Fisher Scientific). Libraries were constructed using NEBNext rRNA Depletion Kit, NEBNext Ultra II Directional RNA Library Prep Kit for Illumina, and NEBNext Multiplex Oligos for Illumina (96 Unique Dual Index Primer Pairs) (NEB). WGBS and RNA-seq libraries were sequenced using the Illumina HiSeq 1500/2500 platform (HCS v2.2.68 and RTA v1.18.66.3) [49] and NovaSeq 6000 platform (NVCS v.1.6 and RTA v.3.4.4).

### Single cell quantitative PCR and microarray analysis

Individual cells obtained from the base of the allantois bud at E7.25-E7.5 were lysed and were subjected directly to cDNA synthesis. Quantitative PCR for *Prdm1* and *Dppa3* expression was performed using the 7900 Real-Time PCR System (Applied Biosystems) [42]. cDNA from individual PGCs was subjected to microarray analysis using GeneChip Mouse Genome 430 2.0 array (Affymetrix) and GeneChip Scanner 3000 (Affymetrix) [50]. Expression levels were calculated as model-based expression index (MBEI) using the dChip 1.3 software (Affymetrix).

### Immunostaining

Growing oocytes collected at postnatal day 7 were fixed in 3.7% paraformaldehyde in PBS for 20 min, washed with PBS containing 0.1% BSA, permeabilized with 0.5% Triton X-100 for 15 min. The cells were denatured with 4 N HCl for 10 min, neutralized with 100 mM Tris-HCl (pH 8.5) for 20 min, and then incubated with 1/500 anti-5mC (Eurogentec) and 1/500 anti-5hmC (Active Motif) primary antibodies for 1 h at room temperature. After washing with in PBS with BSA, the cells were incubated with 1/250 fluorescein isothiocyanate-conjugated anti-mouse IgG (Jackson Immuno-Research) and 1/250 rhodamine-conjugated anti-rabbit IgG (Jackson Immuno-Research) for 1 h. The oocytes were then mounted on a glass slide in VECTASHEILD medium with DAPI (Vector Laboratory) and observed under a CSU-10 confocal laser scanning microscope (Yokogawa) with an ImagEM EM-CCD camera (Hamamatsu).

### Reference sequences

RefSeq transcript assemblies (RefFlat and GTF) and the repeat masker track of mouse genome mm10 were obtained from the UCSC Table Browser [51]. The coordinates of the ICRs [52] were adapted for mm10.

### WGBS data analyses

Reads were trimmed to remove low-quality bases and adapter sequences using Trim-Galore! v0.6.0 (Babraham Institute) and mapped to mouse genome mm10 using Bismark v0.20.0 [53]. Methylation data at CG sites covered with 3–100 reads were extracted for the downstream analyses. Windows with fewer than five informative CG sites were excluded. To study CG methylation at repetitive elements, those with ≥ 50 genomic copies were considered. Differentially methylated regions were defined as 10-kb windows with a CG methylation difference of > 10% and a p value of < 0.05 (t-test). Published WGBS data from epiblast cells, PGCs at E9.5 and E10.5, and control and *Tet1* KO PGCs at E12.5, E13.5, and E14.5 [6, 11, 14] were retrieved from the databases and reprocessed as above.

### RNA-seq data analyses

Reads were trimmed and mapped to mouse genome mm10 by HISAT2 v2.0.5 [54]. Transcripts were assembled by StringTie v2.1.4 [55]. RefSeq-annotated microRNA and snRNA were excluded in the downstream analyses. Genes with (1) fragments per kilobase of exon per million reads mapped (FPKM) ≥ 1 in either E13.5 control or KO female PGCs, (2) > 4-fold changes, and (3) p value of < 0.05 (t-test) were defined as differentially expressed genes. Published RNA-seq data [24] were retrieved and reprocessed for control and maternal KO 2-cell embryos. Expression levels of repetitive elements with ≥ 50 genomic copies were determined using VisR v0.9.42 [57].

### ChIP-seq data analyses

Published ChIP-seq data from E13.5 female PGCs, ESCs and EpiLCs [34, 40, 41] were retrieved. Reads were trimmed and mapped to mouse genome mm10 by bowtie2 v2.2.9 [56]. Duplicate and low-quality reads (MapQ < 5) were removed using Picard v2.6.0 (Broad Institute). Enrichment for repetitive elements with ≥ 50 genomic copies was determined using VisR v0.9.42 [57].

### Statistical analyses and graph generation

Statistical analyses and graph generation were performed by python v3.6.8 [58], deepTools v3.3.1 [59], and the Excel 2016 software (Microsoft). Genome browser shots were generated using Integrative Genomics Viewer [60].

### Electronic supplementary material

Below is the link to the electronic supplementary material.

**Additional file 1**: **Fig. S1.** *Dppa3* KO in mouse PGCs. Related to Fig. 1. **Fig. S2.** CG methylation in oocytes [61, 62]. Related to Fig. 2. **Fig. S3.** Reprogramming defects persists in postnatal oocytes. Related to Fig. 3. **Fig. S4.** DPPA3 acts in the downstream of PRDM14 and independent of TET1. Related to Fig. 4. **Table S1.** Number of PGCs collected in this study. Related to Fig. 1 and Fig. S1. **Table S2.** Sequencing and mapping summary of WGBS. Related to Fig. 1 and Fig. S1. **Table S3.** Sequencing and mapping summary of RNA-seq. Related to Fig. S3. **Table S4.** Downregulated transcripts in maternal KO 2-cell embryos which are persistently hypermethylated in KO PGCs and KO FGOs. Related to Fig. S3

## Data Availability

All raw and processed sequencing data generated in this study have been submitted to the NCBI Gene Expression Omnibus (GEO; https://www.ncbi.nlm.nih.gov/geo/) under accession numbers GSE196620 and GSE203204. The microarray data have been submitted to the NCBI GEO under accession number GSE233342.

## Abbreviations

5hmC: 5-hyroxymethylcytosine
BSA: Bovine serum albumin
EGFP: Enhanced green fluorescent protein
ESC: Embryonic stem cell
FGO: Fully grown oocyte
FPKM: Fragments per kilobase of exon per million reads mapped
H3K9me3: Histone H3 lysine-9 tri-methylation
IAP: Intracisternal A particle
ICR: Imprinting control region
Kb: Kilobase [kb
KO: Knockout (KO)
LTR: Long terminal repeat (LTR)
MBEI: Model-based expression index
PBS: Phosphate-buffered saline
PCR: Polymerase chain reaction
PGC: Primordial germ cell
WGBS: Whole-genome bisulfite sequencing (WGBS)
