# Supplementary material for: DPPA3 facilitates genome-wide DNA demethylation in mouse primordial germ cells
Source: BMC Genomics. 2024 Apr 5;25:344. doi: 10.1186/s12864-024-10192-7 (PMC10996186; doi:10.1186/s12864-024-10192-7)
Supplement: Supplementary file 1 — Additional file 1: Fig. S1. Dppa3 KO in mouse PGCs. Related to Fig. 1. Fig. S2. CG methylation in oocytes [61, 62]. Related to Fig. 2. Fig. S3. Reprogramming defects persists in postnatal oocytes. Related to Fig. 3. Fig. S4. DPPA3 acts in the downstream of PRDM14 and independent of TET1. Related to Fig. 4. Table S1. Number of PGCs collected in this study. Related to Fig. 1 and Fig. S1. Table S2. Sequencing and mapping summary of WGBS. Related to Fig. 1 and Fig. S1. Table S3. Sequencing and mapping summary of RNA-seq. Related to Fig. S3. Table S4. Downregulated transcripts in maternal KO 2-cell embryos which are persistently hypermethylated in KO PGCs and KO FGOs. Related to Fig. S3 [file 12864_2024_10192_MOESM1_ESM.pdf]

Supplementary Information

Additional file 1: Fig. S1-4, Tables S1-4.

Fig. S1.	*Dppa3* KO in mouse PGCs. Related to Fig. 1.
Fig. S2.	CG methylation in oocytes. Related to Fig. 2.
Fig. S3.	Reprogramming defects persists in postnatal oocytes. Related to Fig. 3.
Fig. S4.	DPPA3 acts in the downstream of PRDM14 and independent of TET1. Related to Fig. 4.
Table S1.	Number of PGCs collected in this study. Related to Fig. 1 and Fig. S1.
Table S2.	Sequencing and mapping summary of WGBS. Related to Fig. 1 and Fig. S1.
Table S3.	Sequencing and mapping summary of RNA-seq. Related to Fig. S3.
Table S4.	Downregulated transcripts in maternal KO 2-cell embryos which are persistently hypermethylated in KO PGCs and KO FGOs. Related to Fig. S3.

Fig. S1 | Toriyama et al.

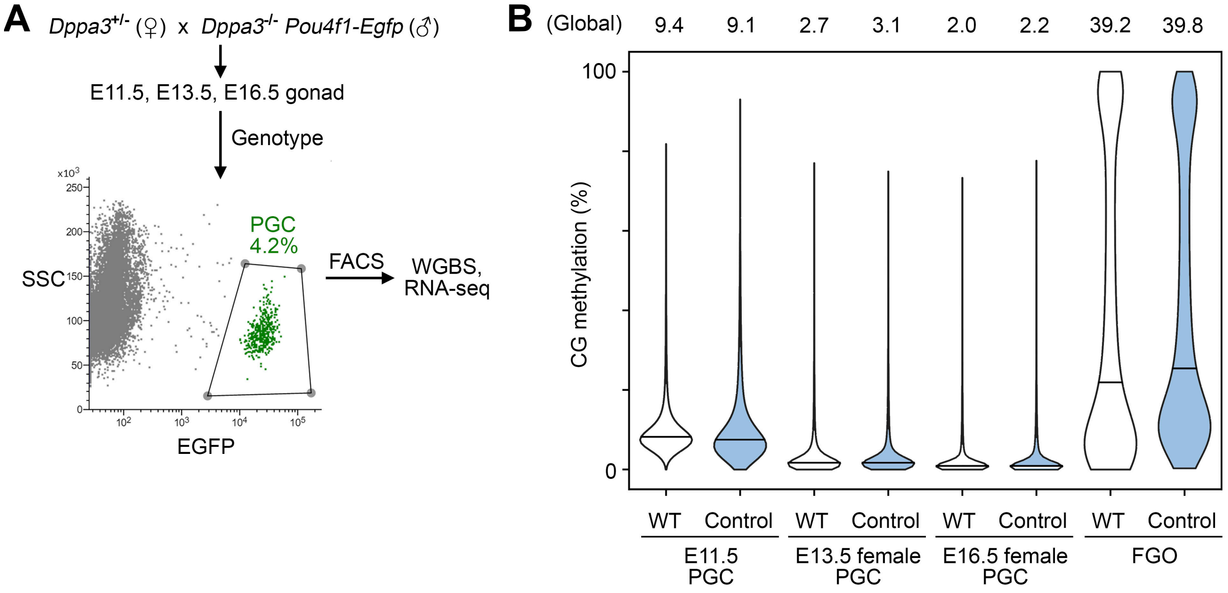

Fig. S1 | *Dppa3* KO in mouse PGCs. Related to Fig. 1. (A) Schematic representation of the mouse cross and PGC collection by flow cytometry using POU5F1-EGFP. Embryos were genotyped, and gonads from the desired genotypes were digested for PGC collection by flow cytometry as EGFP-positive cells (4.2% out of all gonad cells) for WGBS and RNA-seq. Dead and aggregated cells were discarded by monitoring forward and side scatter during sorting. (B) Violin plots showing that the distributions of the regional CG methylation levels (10-kb windows) of wild-type (published) and control PGCs (this study) and those of wild-type and control FGOs (only windows informative in all samples were used, n = 249,088) are essentially the same. Horizontal bars indicate the median values. The global CG methylation levels are indicated above the plots. For wild-type cells, published WGBS data [6,11,31] were retrieved and reprocessed. See also **Additional file 1: Tables S1,2**.

Fig. S2 | Toriyama et al.

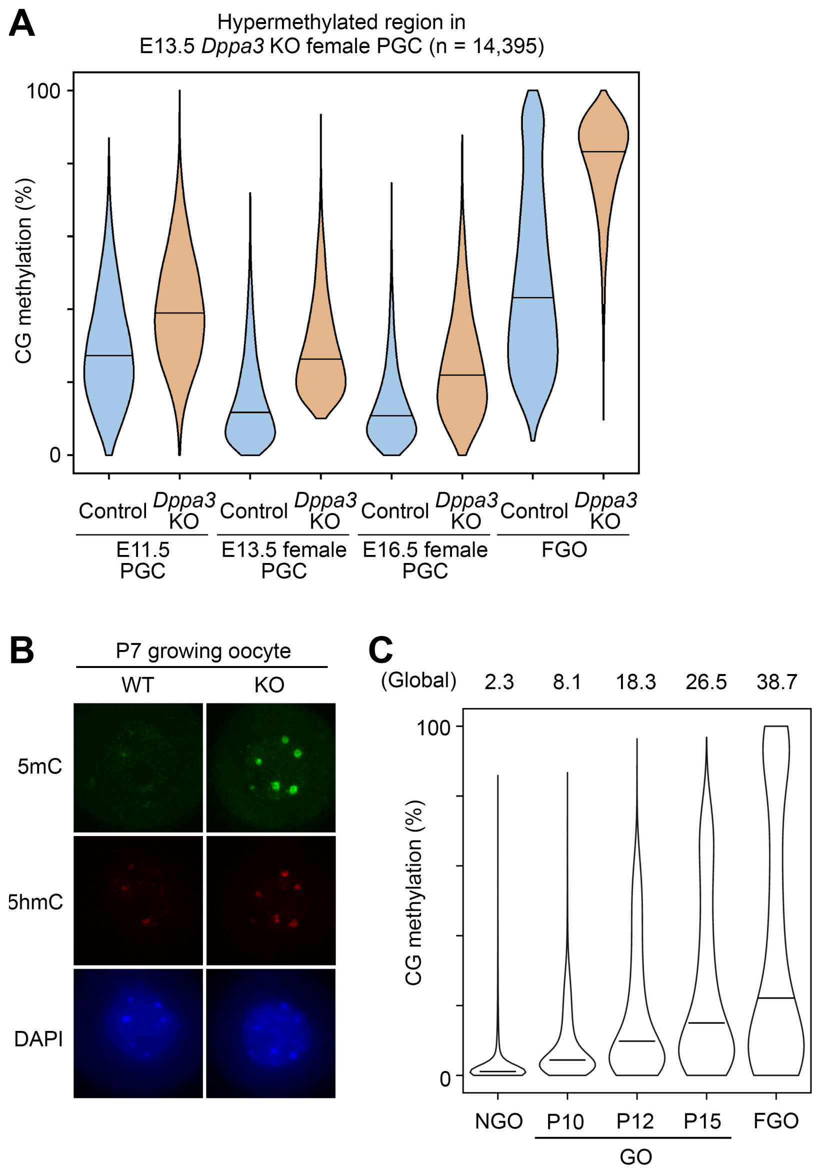

Fig. S2 | CG methylation in oocytes. Related to Fig. 2. (A) Violin plots showing that the CG hypermethylation of *Dppa3* KO PGCs persists in PGCs and FGOs (10-kb windows, n = 14,395). (B) Immunostaining of control and KO GOs at postnatal day 7 for 5mC (green) and 5hmC (red). They were counterstained with DAPI (blue). (C) Violin plots showing the distributions of regional CG methylation levels (10-kb windows) in wild-type NGOs, GOs, and FGOs (only windows informative in all samples were used, n = 272,500). Published WGBS data [16,61,62] were retrieved and reprocessed. Horizontal bars indicate the median values. The global CG methylation levels are indicated above the respective plots.

Fig. S3 | Toriyama et al.

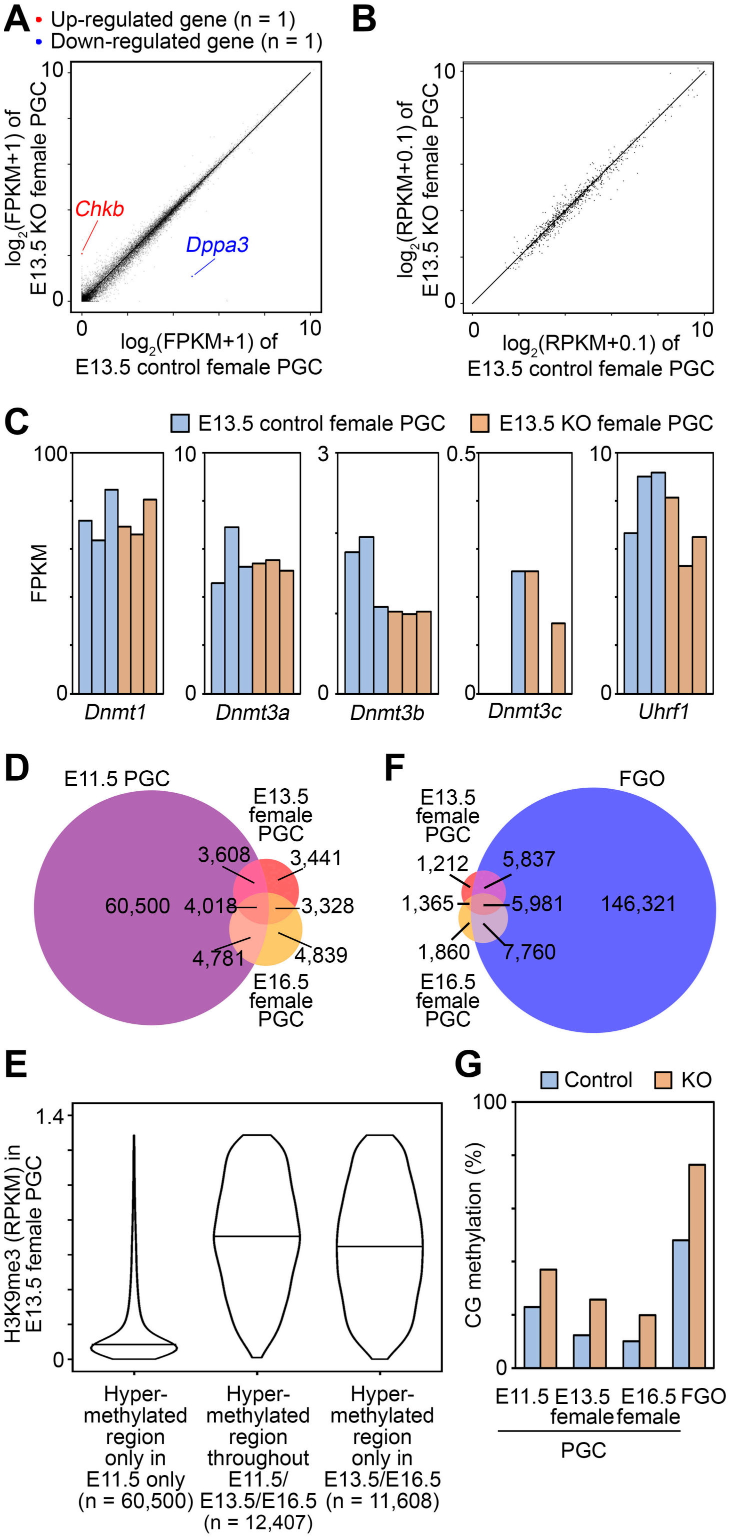

Fig. S3 | Reprogramming defects persists in postnatal oocytes. Related to Fig. 3. (A) Scatterplot comparing the gene expression profiles in E13.5 control and KO female PGCs (n = 24,583). The up- and down-regulated genes are highlighted in red and blue, respectively. (B) Scatterplot comparing the expression levels of repetitive elements in E13.5 control and KO female PGCs (n = 1,033). (C) Gene expression of DNA methyltransferases (*Dnmt1*, *Dnmt3a*, *Dnmt3b*, and *Dnmt3c*) and *Uhrf1* in E13.5 control and KO female PGCs. The FPKM values in all triplicate batches are shown. *Dnmt3l* was not expressed in any batch. (D) Venn diagram showing the overlaps between the hypermethylated regions in E11.5 PGCs and E13.5 and E16.5 female PGCs. The numbers of regions belonging to the respective categories are indicated. (E) Violin plots showing H3K9me3 enrichment in E13.5 wild-type female PGCs for regions hypermethylated only in E11.5, throughout E11.5/E13.5/E16.5 and only in E13.5/E16.5. (F) Venn diagram showing the overlaps between the hypermethylated regions in E13.5 female PGCs, E16.5 female PGCs, and FGOs. The numbers of regions belonging to the respective categories are indicated. (G) CG methylation levels of the major satellite repeat in control and KO PGCs and FGOs.

Fig. S4 | Toriyama et al.

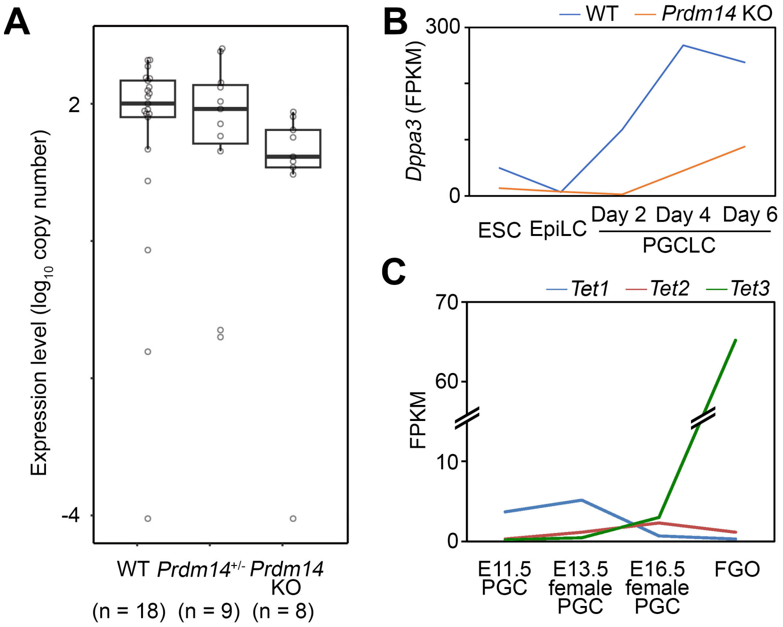

Fig. S4 | DPPA3 acts in the downstream of PRDM14 and independent of TET1. Related to Fig. 4. (A) *Dppa3* expression in E7.25-E7.5 *Prdm14* KO PGCs using single cell quantitative PCR analysis. (B) *Dppa3* expression during *in vitro* specification of PGCLCs. Published RNA-seq data from wild-type and *Prdm14* KO PGCLCs [43] were reprocessed. (C) *Tet* family expression during PGC development and oogenesis. Published RNA-seq data from wild-type PGCs [6] and FGOs [44] were reprocessed.

Table S1. Number of PGCs collected in this study. Related to Fig. 1 and Fig. S1

	No. of PGCs collected	
	Control	*Dppa3* KO
E11.5 PGC	86	28
		30
E13.5 female PGC	342	689
	517	1375
	352	1012
	165	363
	746	642
E16.5 female PGC	427	1032

Table S2. Sequencing and mapping summary of WGBS.
Related to Fig. 1 and Fig. S1

Sample		Total number of reads	Uniquely mapped reads	Mapping efficiency (%)	Average depth per strand	Bisulfite conversion rate (%)	Global CG methylation (%)	Correlation coefficient of CG methylation (in 500-kb windows)
E11.5 control PGC		1,044,130,842	446,778,418	60.4	18.3	99.3	9.1	
E11.5 *Dppa3* KO PGC	rep 1	1,009,735,310	162,223,015	23.8	6.9	99.0	16.3	0.93
	rep 2	997,898,274	198,091,799	28.4	8.1	99.2	14.7	
E13.5 control female PGC	rep 1	1,023,871,003	416,349,986	58.8	17.6	99.5	3.0	0.97
	rep 2	1,013,551,287	361,772,971	55.7	15.4	99.2	3.3	
E13.5 *Dppa3* KO female PGC	rep 1	1,036,150,184	441,962,069	60.5	18.7	99.5	4.8	0.98
	rep 2	1,038,062,764	373,934,327	56.0	15.9	99.2	4.8	
E16.5 control female PGC		939,961,978	340,380,379	58.4	14.5	99.5	2.2	
E16.5 *Dppa3* KO female PGC		912,996,500	370,137,977	62.0	15.7	99.4	3.8	
Control FGO	rep 1	676,854,754	346,524,059	61.7	12.7	99.5	39.9	0.99
	rep 2	221,016,789	115,846,797	58.4	4.1	99.4	39.9	
Dppa3 KO FGO	rep 1	911,463,298	484,732,946	62.5	17.4	99.5	64.4	0.99
	rep 2	195,280,108	103,500,944	60.4	3.7	99.4	63.6	

Table S3. Sequencing and mapping summary of RNA-seq. Related to Fig. S3

Sample		Total number of reads	Uniquely mapped reads	Mapping efficiency (including multiple mapped reads) (%)	Mapping efficiency (only uniquely mapped reads) (%)	Correlation coefficient (in FPKM)
E13.5 control female PGC	rep 1	84,896,636	27,993,344	45.7	37.9	>0.97
	rep 2	78,523,400	28,408,480	44.2	36.4	
	rep 3	74,431,772	32,131,764	46.5	38.1	
E13.5 *Dppa3* KO female PGC	rep 1	84,994,002	32,522,818	47.0	38.6	>0.99
	rep 2	96,905,632	34,243,768	53.3	35.6	
	rep 3	107,116,304	40,150,414	45.8	37.7	

Table S4. Downregulated transcripts in maternal KO 2-cell embryos which are peristently hypermethylated in KO PGCs and KO FGOs. Related to Fig. S3

GeneName	Expression (FPKM)		Description
	Control	*Dppa3* mat-KO	
Olfr1465	0.56	0.00	Olfactory receptor
Olfr348	0.76	0.00	
Olfr494	0.69	0.00	
Olfr504	0.61	0.00	
Olfr589	0.54	0.00	
Olfr617	0.68	0.00	
Olfr663	0.50	0.00	
Olfr963	0.55	0.00	
Vmn1r158	0.91	0.00	
Vmn1r234	0.52	0.00	
Vmn1r61	0.50	0.00	
Taar7d	1.07	0.00	Grueneberg ganglion trace-amine receptor,
Cypt1	2.17	0.68	Acrosome-localized cysteine-rich perinuclear theca protein
Mrgpra1	0.83	0.00	Mast cell G protein membrane protein
Gm19757	9.16	0.72	Unannotated gene
